# Impulse Noise Induced Hidden Hearing Loss, Hair Cell Ciliary Changes and Oxidative Stress in Mice

**DOI:** 10.3390/antiox10121880

**Published:** 2021-11-25

**Authors:** Paul Gratias, Jamal Nasr, Corentin Affortit, Jean-Charles Ceccato, Florence François, François Casas, Rémy Pujol, Sylvie Pucheu, Jean-Luc Puel, Jing Wang

**Affiliations:** 1 Institute for Neurosciences of Montpellier (INM), University Montpellier, INSERM, 34091 Montpellier, France; paul.gratias@inserm.fr (P.G.); jamal.nasr@inserm.fr (J.N.); corentin.affortit@inserm.fr (C.A.); jean-charles.ceccato@umontpellier.fr (J.-C.C.); florence.francois@inserm.fr (F.F.); remy.pujol@inserm.fr (R.P.); Jean-luc.puel@inserm.fr (J.-L.P.); 2 Unité Dynamique Du Muscle et Métabolisme (DMEM), Institut National de Recherche pour l’Agriculture, l’Alimentation et l’Environnement (INRAE), University Montpellier, 34060 Montpellier, France; francois.casas@inrae.fr; 3 Cilcare, 371 Rue du Professeur J. Blayac, 34080 Montpellier, France; sylvie.pucheu@cilcare.com; 4 ENT Department, Hospital and University of Montpellier, 34091 Montpellier, France

**Keywords:** impulse noise, oxidative stress, hidden hearing loss, cochlea

## Abstract

Recent studies demonstrated that reversible continuous noise exposure may induce a temporary threshold shift (TTS) with a permanent degeneration of auditory nerve fibers, although hair cells remain intact. To probe the impact of TTS-inducing impulse noise exposure on hearing, CBA/J Mice were exposed to noise impulses with peak pressures of 145 dB SPL. We found that 30 min after exposure, the noise caused a mean elevation of ABR thresholds of ~30 dB and a reduction in DPOAE amplitude. Four weeks later, ABR thresholds and DPOAE amplitude were back to normal in the higher frequency region (8–32 kHz). At lower frequencies, a small degree of PTS remained. Morphological evaluations revealed a disturbance of the stereociliary bundle of outer hair cells, mainly located in the apical regions. On the other hand, the reduced suprathreshold ABR amplitudes remained until 4 weeks later. A loss of synapse numbers was observed 24 h after exposure, with full recovery two weeks later. Transmission electron microscopy revealed morphological changes at the ribbon synapses by two weeks post exposure. In addition, increased levels of oxidative stress were observed immediately after exposure, and maintained for a further 2 weeks. These results clarify the pathology underlying impulse noise-induced sensory dysfunction, and suggest possible links between impulse-noise injury, cochlear cell morphology, metabolic changes, and hidden hearing loss.

## 1. Introduction

Noise-induced hearing loss (NIHL) acquired in leisure or occupational settings is a common cause of hearing impairment in industrialized countries, with a prevalence second only to age-related hearing loss (ARHL) [1,2]. It is well known that after high-intensity exposure, an irreversible increase in hearing thresholds can occur, leading to a permanent threshold shift (PTS) due to the loss of cochlear sensory hair cells [3]. Hearing loss associated with mild acoustic overexposure is reversible, and hearing recovers within 2–3 weeks [4]. This temporary loss is known as a temporary threshold shift (TTS), and is probably due to reversible damage to the stereocilia of hair cells [5] and/or swelling, followed by recovery, of cochlear nerve terminals [6,7].

Some recent studies demonstrated that TTS exposure may cause the loss of more than 50% of the synapses that lie between the cochlear nerve fibers and inner hair cells (IHCs), without hair-cell damage and without alteration in hearing thresholds [8,9]. This selective synaptopathy occurring after noise exposure was thus named “hidden hearing loss” [10]. The threshold recovery to normal levels was attributed to the recovery of OHC function, together with the unexpected resilience of the high-spontaneous-rate auditory fibers encoding the best thresholds. Nevertheless, the fragility of the low-spontaneous rate fibers is not yet understood. Cochlear synaptopathy might contribute to impairment of the ability to understand speech in loud background noise [11], and also to hyperacusis and/or tinnitus [12,13]. Finally, the synaptopathy induced by noise exposure would contribute to the early onset of neural age-related hearing loss in mice [14,15].

However, to date most findings from studies in animal models have demonstrated cochlear synaptopathy and neurodegenerative processes apparently linked to continuous octave-band noise exposure at sound pressure levels of ~100 dB SPL for ~2 h [10,11,14,16]. Only a recent study in blast-noise-exposed chinchillas [17] showed that the synapses between inner hair cells and dendrites of spiral ganglion neurons are most vulnerable to blast exposure of 165 dB SPL peak.

Impulses noises, resulting from the sudden release of energy into the atmosphere (explosions, gunshot) or from impacts between objects (machine tools, hammering) are common in industrial activity, construction and military [18,19,20]. Perceptual anomalies such as tinnitus or hyperacusis, and difficulty hearing in noise, often occur and persist after blast-induced damage, even in cases where threshold sensitivity has returned to normal [21,22,23]. Surprisingly, although there is a growing body of knowledge documenting the effects of continuous noise exposure on hearing PTS or TTS [10,11,14,16,24,25,26,27,28], there is very little literature on the effects of blast-wave and impulse noise-exposure on hearing function.

The purpose of this study was to probe cochlear function, morphology and metabolic state after a moderate impulse noise exposure to determine whether, as for continuous-noise exposures, the synaptic connections between hair cells and cochlear nerve fibers are more vulnerable than the hair cells themselves to impulse-noise exposures. To do so, awake mice were exposed to an impulse noise with peak pressures of 146 dB SPL. The effects of this exposure were assessed using complementary approaches combining morpho-physiology, biochemistry and molecular biology. 

## 2. Materials and Methods

### 2.1. Animals

Male CBA/J mice were purchased from Janvier Laboratories (Le Genest-Saint-Isle, France) and were housed in facilities accredited by the French Ministry of Agriculture and Food (D-34-172-36; 20 May 2021). Experiments were carried out in accordance with French Ethical Committee stipulations regarding the care and use of animals for experimental procedures (agreements C75-05-18 and 01476.02, license #6711). All experimental procedures were conducted with 10–14-week-old male mice. All efforts were made to minimize the number of animals used and their suffering.

### 2.2. Impulse Exposures

**Figure 1:**
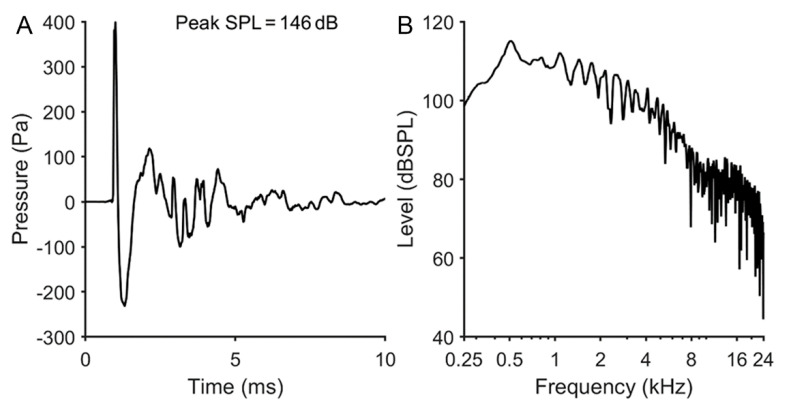
Example of impulse noise waveforms measured at the animal’s head. (**A**) Pressure-time waveform obtained from one impulse at a peak pressure of 146 dB SPL. (**B**) Energy spectrum of the 10 ms pressure-time waveform shown in (**A**).

Awake mice were placed, singly and unrestrained, in a small wire mesh cage suspended directly below the acoustic horn of a loudspeaker that extended into an exposure chamber lined with acoustic foam to reduce sound reverberation. The explosion-like impulses were generated by a customized system. Briefly, the noise was generated by a PCI 4461 card (National instruments, Austin, TX, USA) using the Friedlander equation in LabVIEW as described by Qin et al., 2015 [29]. To measure the impulse noise generated, a ¼” high-sensitivity condenser microphone set (GRAS 46BF) was used. The microphone was aligned at the center of the horn and the impulse noise generated at different output voltages (from 0.3 to 8 V). A pilot study was performed to obtain a temporary threshold shift (TTS) without visible eardrum rupture by varying the intensity and the rate of presentation. The best compromise was a presentation of 700 broad-spectrum (0.25–24 kHz) impulses with peak SPL of 145 ± 0.5 at 1 Hz pulse repetition rate (total duration: 11 min 40 s) (Figure 1).

### 2.3. Functional Hearing Assessments

Functional evaluation of ears was performed by recording auditory brainstem responses (ABRs) and distortion-product otoacoustic emissions (DPOAEs) in anesthetized mice before, 30 min, 1 day and 2 and 4 weeks after noise exposure (*n* = 11 animals, 22 cochleae for each time point). All functional evaluations were carried out in a Faraday-shielded, anechoic soundproof cage. Rectal temperature was measured with a thermistor probe, and maintained at 38.5 ± 1 °C using an underlying heated blanket. For evaluation of age-related hearing loss, 4 age-matched additional mice (*n* = 8 cochleae) were recorded at 14 weeks of age and at 18 weeks of age (see Supplementary Figure S1).

#### 2.3.1. Distortion-Product Otoacoustic Emission (DPOAEs)

DPOAEs were recorded in the external auditory canal using an ER-10C S/N 2528 probe (Etymotic research Inc. Elk Grove Village, IL, USA.). Stimuli were two equi-level (65 dB SPL) primary tones, f1 and f2, with a constant f2/f1 ratio of 1.2. The DPOAE 2f1-f2 was extracted from the ear canal sound pressure and processed by a HearID auditory diagnostic system (Mimosa Acoustic, Champaign, IL USA) on a computer. The probe was self-calibrated for the two stimulating tones before each recording. f1 and f2 were presented simultaneously, stepping f2 from 20 to 20 kHz in quarter-octave steps. For each frequency, the distortion product 2f1-f2 and the neighboring noise amplitude levels were measured and expressed as a function of f2.

#### 2.3.2. Auditory Brainstem Response (ABRs)

ABRs were recorded using three subcutaneous needle electrodes placed on the vertex (active), on the pinna of the tested ear (reference) and in the hind leg (ground). Strong correlations were observed between click-evoked ABR thresholds and pure-tone thresholds at 2 and 4 kHz [30]. To obtain more frequency-specific estimates of hearing sensitivity in the high-frequency range, we chose to use tone-burst stimulation for ABR recording. Sound stimuli were generated by a NI PXI-4461 signal generator (National Instruments) and consisted of 9 ms tone bursts, with a 7 ms plateau and 1 ms rise/fall times, delivered at a rate of 11/s with alternate polarity by a JBL 2426H loudspeaker in a calibrated free field. Stimuli were presented to the ear by varying levels from 100 to 0 dB SPL, in 5 dB steps. Stimuli were generated and data acquired using Matlab (MathWorks, Natick, MA, USA) and LabView (National Instruments) software. The difference potential between vertex and mastoid intradermal needles was amplified (20,000 times, Grass P511 differential amplifier), sampled (at a rate of 50 kHz), filtered (bandwidth of 0.3–3 kHz) and averaged (700 times). Data were displayed using LabView software and stored on a computer (Dell precision 3630). ABR thresholds were defined as the lowest sound level that elicited a clearly distinguishable wave II. Recordings and analysis were performed blindly.

### 2.4. Morphological Assessments

Eighteen additional mice were used for the assessment of noise-induced ultrastructural changes in sensory neural cells of the cochlea using scanning (SEM, Hitachi S4000) and transmission electron microscopy (TEM, Tecnai F20 FEI 200KV). In addition, 24 additional mice were required for counting of ribbon synapses of IHCs and immunocytochemistry using confocal microscopy.

#### 2.4.1. Counting of Sensory Hair Cells 

Sensory hair-cell loss was evaluated using SEM. The cochleae were processed and evaluated using previously reported standard techniques [31]. Counting of inner (IHC) and outer (OHC) hair cells was performed in three different 300 µm-long segments of the organ of Corti located at 0.5 to 1, 1.1 to 2.5, and 2.6 to 3.7 mm from the apex tip, corresponding to the 6 to 8, 8 to 16 and 16 to 25 kHz regions, respectively, from both control and noise-exposed cochleae with different post-exposure times (*n* = 4 to 6 cochleae per time point). Hair cells were considered to be absent if the stereociliary bundles and cuticular plates were missing [31,32]. Disruption of the OHC stereociliary bundle was defined as bending at their base of the outermost row of stereocilia toward the lateral side, while the other bundle rows remained straight. To minimize bias, two different experimenters performed the counts.

#### 2.4.2. Ultrastructural Analysis

Morphological damage related to noise exposure was investigated using TEM of the basal cochlear region. Animals were decapitated under deep anesthesia, and their cochleae were prepared according to a standard protocol for fixation and plastic embedding. Semi-thin sections were observed under a Zeiss Axioscope light microscope, and ultrathin radial sections of the organ of Corti were analyzed using TEM (*n* = 5–6 cochleae per time point).

#### 2.4.3. Counting of the Auditory Nerve-Fiber Terminals 

The density of the auditory nerve-fiber terminals was measured in 3 to 4 habenular openings of the cochlear semi-thin sections of the osseous spiral from the cochlear regions coding between 16 and 25 kHz of control and noise-exposed mice at 2 weeks after. The mean value of each section was then averaged for each animal and each group (*n* = 3 sections per animal, 4–5 cochleae per group).

#### 2.4.4. Counting of Ribbon Synapses of IHCs

Immunocytochemistry was performed in mouse cochlear whole-mount preparations before, 24 h and 2 weeks (*n* = 7–8 cochleae for each time point) after noise exposure. The cochlear samples were immunostained with anti-CtBP2 (1:500, BD Transduction Laboratories, Le Pont-de-Claix, France, Cat #612044 RRID:AB_399431) and anti-Homer1 (1:500, GeneTex, Irvine, CA, USA, #GTX103278, RRID: AB_1950505) to label the presynaptic and postsynaptic structures, respectively. The samples were counterstained with anti-Vglut3 (1/500, Synaptic Systems, Göttingen, Germany, Cat #135204 RRID:AB_2619825) to label the inner hair cells. All secondary antibodies were used at a dilution of 1:1000. These included donkey anti-mouse and anti-rabbit, and goat anti-guinea pig IgG conjugated to Alexa 488, Alexa 594 or Alexa 647, respectively (Molecular Probes, Eugene, OR, USA, Cat #A-21202 RRID:AB-141607, Cat # A-21207, RRID:AB_141637, Cat # A-21450 RRID:AB_141882). Fluorescent tags were visualized using a confocal microscope (Zeiss 880 Airyscan). In control specimens without primary antibodies, neither Alexa 488, 594 nor 647 fluorescent tags were observed. The quantitative analyses were performed in the area between 6 and 45 kHz. Colocalization of synaptic punctum was assessed using Imaris (Version 9.5, Bitplane). CtBP2 and Homer1 puncta were detected using the spot algorithm allowing for different spot sizes and elongated point spread function in z-direction (initial seed size x − y/z, Ctbp2, Homer1, 0.55/1.1 µm; followed by colocalizing the spots with a threshold lower than 1.

### 2.5. Molecular Assessment

#### 2.5.1. Enzymatic Activities and Lipid Peroxidation

Cochlear homogenates were prepared as described by Casas [33] and the protein concentration measured using the Bradford method. Lipid peroxidation was assessed using the thiobarbituric acid-reactive substances method, and expressed in nmol/mg malondialdehyde (MDA) [33]. Catalase and SOD activities were measured as described, respectively, by Beers and Sizer [34] and Marklund [35]. Complex I, complex II and COX activities were measured as described previously [36,37,38] and expressed in mU/mg protein. Enzymatic activities and lipid-peroxidation analysis required 8 additional animals (16 cochleae) per time point. All experiments were performed in triplicate.

#### 2.5.2. Immunocytochemistry 

Immunocytochemistry in cryostat sections was employed to probe the potential impact of noise exposure on the integrity of mitochondrial complexes as well as the cellular localization of oxidative stress and cellular-survival markers. The primary antibody used was that recognizing SOD2 (1/500, Abcam, Cambridge, UK, Cat #ab13533, RRID:AB_300434). Anti-neurofilament (NF 200, 1/600, Sigma-Aldrich, Saint-Lois, MO, USA Cat#N0142, RRID:AB_477257) was used to identify the spiral ganglion neurons, Alexa 594 phalloidin (1/1000, Thermo Fisher Scientific, Waltham, MA, USA, Cat# A12381, RRID:AB_2315633) to label actin, and Hoechst 33342 (0.002% wt:vol, Sigma-Aldrich, Saint-Lois, MO, USA) to stain the nuclei. All secondary antibodies were used at a dilution of 1/1000. This included donkey anti-mouse and anti-rabbit IgG conjugated to Alexa 488 or Alexa 594 (Molecular Probes, Eugene, OR, USA, Cat #A-21202, RRID:AB_141607; Molecular Probes, Eugene, OR, USA, Cat #A-21206, RRID:AB_2535792; Molecular Probes, Eugene, OR, USA, Cat# A-21203, RRID:AB_141633; Molecular Probes, Eugene, OR, USA, Cat# A-21207, RRID:AB_141637). Fluorescent tags were visualized using a confocal microscope (Zeiss 880 Airyscan). In control specimens without primary antibodies, neither Alexa 488 nor 594 fluorescent tags were observed. Immunocytochemistry analysis required 4 to 5 additional cochleae per age and strain. All experiments were performed in triplicate.

### 2.6. Statistics 

Data are expressed as the mean ± SEM, statistical analyses were carried out using GraphPad Prism8 (GraphPad, San Diego, CA, USA). Normality of the variables was assessed using the Shapiro–Wilks test. The significance of the group differences for normal data was assessed with a two-way ANOVA followed by Dunnett’s multiple comparisons test. If data failed to pass the normality test, a Friedman ANOVA followed by Dunn’s multiple comparison test was used. Comparisons were made with the control condition, or between before the noise exposure and multiple times after noise exposure. The level of statistical significance was set to be *p* ≤ 0.05.

Based on data from our previous reports [39] or from preliminary experiments, we calculated the sample size using G*Power 3.1.9.2 to ensure adequate power of key experiments for detecting prespecified effect sizes.

## 3. Results

### 3.1. Impulse Noise Induced Reversible Threshold Shifts at the Higher Frequencies 

**Figure 2:**
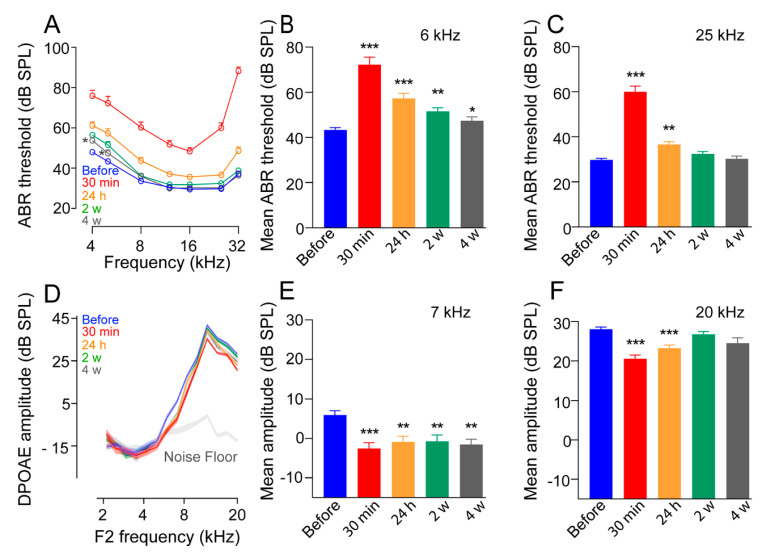
Broad-spectrum impulse noise-induced temporary threshold shift. (**A**,**D**): ABR thresholds (**A**) and DPOAE amplitude (**D**) recorded before (blue) and 30 min (red), 24 h (orange), 2 weeks (green), and 4 weeks (gray) after impulse-noise exposure. Note complete recovery of ABR thresholds and DPOAE amplitude in the higher-frequency regions (8 to 32 and 8 to 20 kHz, for ABR and DPOAE, respectively) by 2 and 4 weeks after exposure. (**B**,**C**): Mean ABR thresholds at 6 to 32 kHz for all evaluation time points. (**E**,**F**): Mean DPOAE amplitudes at 6 to 32 kHz for all time points. All data are expressed as mean ± SEM (*n* = 22 cochleae per time point); two-way ANOVA test was followed by Dunnett’s multiple comparison: * *p* ≤ 0.05, ** *p* ≤ 0.01, *** *p* ≤ 0.001, time after noise exposure vs. before.

We first evaluated the impact of impulse noise exposures on hearing function. Our results showed that 30 min after exposure, mice displayed an elevation of ABR thresholds of ≥30 dB for all frequencies (Figure 2A). During the first 24 h following the noise exposure, there was a partial recovery of ABR thresholds of around 20 dB. Four weeks after, complete recovery of ABR thresholds was observed at the higher frequencies (8 to 32 kHz), but not the lower frequencies (4 and 6 kHz), where <5 dB PTS still remained (Figure 2A). These results were confirmed by the mean ABR thresholds at 6 and 25 kHz, that showed a significant increase in the ABR thresholds 30 min after impulse-noise exposure. Complete recovery was observed in the 25 kHz region from 2 weeks, but not at 6 kHz, where a slight but significant elevation in ABR thresholds was still observed 4 weeks after exposure (*p* < 0.05, Figure 2B,C).

### 3.2. Alteration of Distortion-Product Otoacoustic Emissions

OHCs act as nonlinear feedback amplifiers that enhance the sensitivity and the frequency selectivity of the hearing organ. DPOAEs are the by-product of this nonlinear amplification process and hence can serve as a measure for evaluating the integrity of OHCs. Thirty minutes following noise exposure, a moderate decrease in the amplitude of DPOAEs in the frequency range from 6 to 20 kHz was observed, returning to nearly pre-exposure levels at the higher frequencies two weeks later (Figure 2D). These results were confirmed by the mean DPOAE amplitude evoked by frequencies at 7 and 20 kHz. Significantly (*p* < 0.01) reduced amplitude of DPOAEs was observed at 7 kHz until 4 weeks after exposure, while complete recovery of DPOAE amplitude was observed at 20 kHz (Figure 2E,F) from 2 weeks after exposure. These results suggest impulse noise induced impaired sound processing at lower frequencies in the cochlea.

### 3.3. Reduced ABR Wave-I Amplitude and Elevated Central Gain following Exposure 

Continuous noise exposures produce robust TTS and permanently reduced ABR wave-I amplitudes at supra-threshold levels in mice, together with degeneration of low-spontaneous-rate auditory nerve fibers [14]. Here, 30 min after impulse-noise exposure, ABR wave-I amplitudes elicited by 4, 8, 16 and 32 kHz tone-burst stimulation were dramatically reduced at all sound levels tested, and more importantly in the 32 kHz region. These results suggest impulse noise induced acute dysfunction of OHCs and cochlear synaptopathy. A progressive partial recovery of ABR wave-I amplitudes was observed from 24 h to 4 weeks following noise exposure at all sound levels and for all frequencies. At 4 weeks after noise exposure, although ABR thresholds had completely recovered to pre-exposure levels at the higher frequencies (8 to 32 kHz, Figure 2A–C), the amplitudes of wave I of the ABR remained lower at all sound levels tested and from low to high frequencies compared to pre-exposure values.

**Figure 3:**
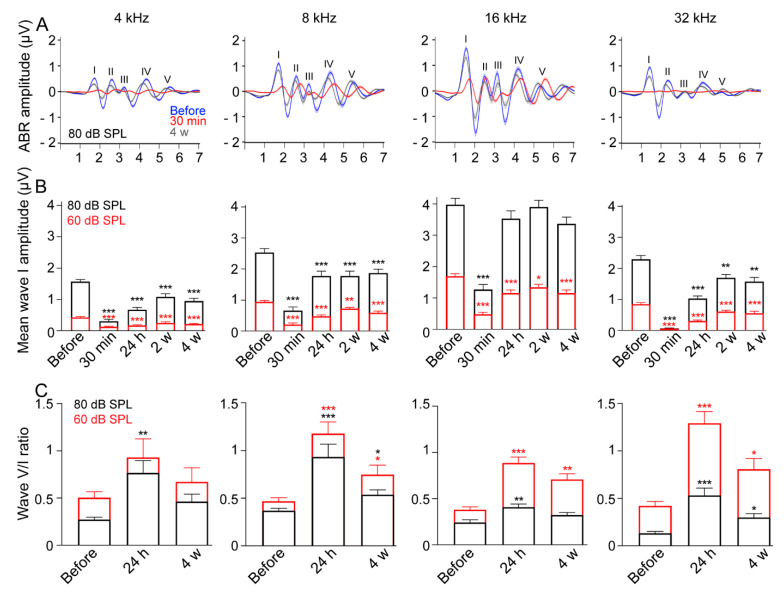
Impulse-noise effect on the amplitude of ABR waves. (**A**): Mean ABR waveforms evoked by 4, 8, 16 and 32 kHz tone bursts at 80 dB SPL, and recorded before (blue), 30 min (red), and 4 weeks (gray) after impulse-noise exposure. (**B**): ABR Wave I amplitudes evoked by 4, 8, 16 and 32 kHz tone bursts at 60 dB SPL (red), and 80 dB SPL (black), and recorded before, 30 min, 24 h or 4 weeks after impulse-noise exposure. Two-way ANOVA test was followed by Dunnett’s multiple comparison: * *p* ≤ 0.05, ** *p* ≤ 0.01, *** *p* ≤ 0.001, time after noise exposure vs. before. (**C**): Mean ABR Wave V/I amplitude ratios recorded before, 24 h, or 4 weeks after impulse noise-exposure at 60 dB SPL (red), or 80 dB SPL (black). ABR waves V/I ratio failed to pass the normality test, so Friedman ANOVA was followed by Dunn’s multiple comparison test: * *p* ≤ 0.05, ** *p* ≤ 0.01, *** *p* ≤ 0.001, time after noise exposure vs. before. All data are expressed as mean ± SEM (*n* = 22 cochleae per time point).

The average ABR waveforms elicited by all tone-burst frequencies at 80 dB SPL (Figure 3A) showed strong reduction in the amplitude of all waves, except for wave V elicited by 16 kHz at 30 min following impulse noise exposure. Four weeks after exposure, while an almost complete recovery in the amplitude of wave V and peaks I and II still remained smaller than pre-exposure (Figure 3A), suggesting that a compensatory mechanism might have affected central processing, mean ABR amplitudes elicited by 4, 8, 16 and 32 kHz tone-bursts at 60 (Figure 3B, red plots) and 80 dB SPL (Figure 3B, black plots), showed a significant reduction in wave I amplitudes until 4 weeks after exposure (Figure 3B), except for 16 kHz at 80 dB SPL stimulus, where a complete recovery was seen after 24 h (Figure 3B).

In addition, a significant increase in the V/I wave ratio was observed for all tone-burst frequencies at 60 or 80 dB SPL stimulus by 24 h, and maintained to 4 weeks after exposure for 8, 16 and 32 kHz (Figure 3C). The differences between before and 4 weeks after exposure at 60 or 80 dB SPL stimulus for 4 kHz, and for 16 kHz at 60 dB SPL stimulus, however, were not significant (*p* > 0.05, Figure 3C).

Together, these results suggest that impulse noise induced permanent, moderate OHC dysfunction, together with reduced ABR wave-I amplitudes at supra-threshold levels. In addition, there was evidence of compensatory mechanisms in central processing. 

### 3.4. Disturbances in Stereociliary Bundle Morphology of the Outer Hair Cells

**Figure 4:**
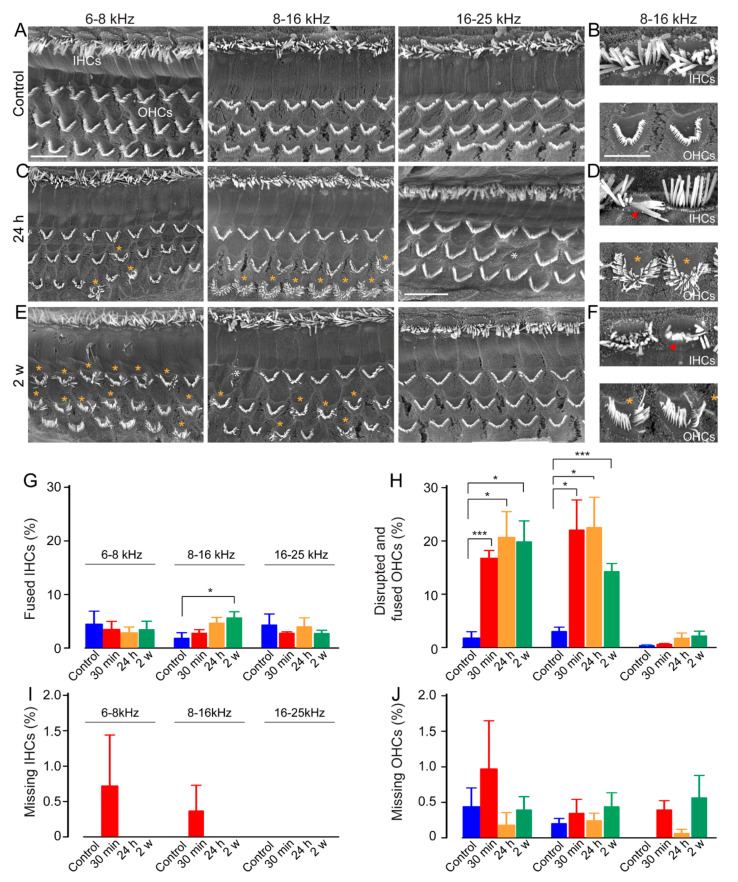
Alteration of the OHC stereocilia A, C and E: Representative scanning electron microscopy from different coding regions of cochleae (6–8 kHz, 8–16 kHz, 16–25 kHz) from control (**A**), 24 h (**C**), and 2 weeks (**E**) after exposure. (**B**,**D**,**F**): Higher-magnification images of representative hair bundles of the OHCs located in the region coding 8–16 kHz. Yellow and white asterisks indicate disrupted and fragmented hair bundles of the OHCs and loss of OHC, respectively. Red asterisks pinpoint fused stereocilia of the IHCs. Scale bars: 15 μm. (**G**–**J**): Cytocochleograms representing the percentage of fused IHCs (**G**) disrupted and fused OHCs (**H**), missing IHCs (**I**), and missing OHCs (**J**) in different coding regions (6–8, 8–16, 16–25 kHz) from control (blue bars), 30 min (red bars), 24 h (orange bars) and 2 weeks (green bars) after exposure. Data are expressed as mean ± SEM (*n* = 6 to 12 cochleae per age and genotype). One-way ANOVA test was followed by Dunn’s test. * *p* ≤ 0.05, *** *p* ≤ 0.001, vs. control.

To assess the effects of impulse-noise exposure on the hair cells, we performed scanning electron microscopy (SEM), which allows the visualization of the surface of the organ of Corti. Twenty-four hours after exposure, disturbance of stereocilial morphology was observed mainly in outer hair cells located in the regions coding the frequencies from 4 to 16 kHz that matched the changes in the DPOAE (Figure 4C,D), compared to a normal appearance of the hair cell bundles in control unexposed cochleae (Figure 4A,B). The bundle disruption did not recover until 15 days after exposure (Figure 4E,F). In addition, a few IHCs also showed fused stereocilia (Figure 4D,F). 

Quantification analysis revealed that a significantly higher number of OHCs had disrupted or fused hair bundles in the cochlear region coding 6 to 16 kHz in noise-exposed mice by 30 min, and maintained to 2 weeks after exposure (*p* < 0.05 vs. before, Figure 4H). A slight but significant increase in IHCs with fused hair bundles was also observed in the 8–16 kHz coding region of the exposed mice at 2 weeks after (*p* < 0.05 vs. before, Figure 4G). By contrast, no significant loss of IHCs and OHCs was observed in either control unexposed or noise-exposed mice (Figure 4I,J).

### 3.5. Reversible and Moderate Loss of IHC Ribbon Synapses

**Figure 5:**
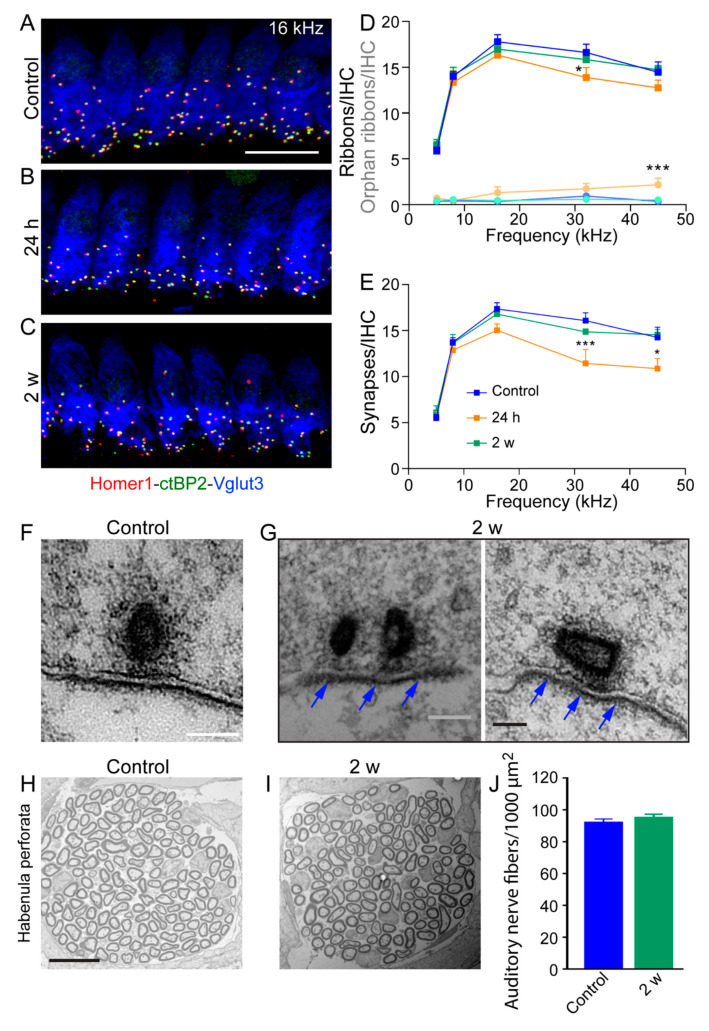
Reversible and moderate loss of IHC ribbon synapses. (**A**–**C**): Maximum intensity projections of confocal z-stacks of inner hair cells (IHCs) immunolabeled with anti-Vglut3 (blue), anti-Homer1 (red) and anti-CtBP2 (green) in the mid-cochlear region from control mice (**A**), noise-exposed mice at 24 h (**B**), and 2 weeks after (**C**). Scale bar = 10µm. (**D**): Quantifications of ribbons total (all CtBP2 puncta, blue, green and orange lines) and orphan ribbons (unpaired CtBP2 puncta, light blue, green and orange lines) per IHC along the tonotopic axis of the cochleae from control (blue), and impulse noise-exposed mice at 24 h (orange) and 2 weeks (green) after exposure. Two-way ANOVA test was followed by Dunnett’s multiple comparison: * *p* ≤ 0.05, time after noise exposure *vs.* before. All data are expressed as mean ± SEM (*n* = 7–8 cochleae for per time point). (**E**): Quantifications of synapses (paired CtBP2-Homer1 puncta). Two-way ANOVA test was followed by Dunnett’s multiple comparison: * *p* ≤ 0.05, *** *p* ≤ 0.001, time after noise exposure vs. before. All data are expressed as mean ± SEM (*n*= 7–8 cochleae for per time point). (**F**,**G**): TEM images of ribbon synapses from the cochlear regions coding 16–25 kHz from control (**F**), and 2 weeks (**G**) after exposure. Note, at 2 weeks after exposure (**G**), some immature synapse morphology: a double presynaptic ribbon or a disoriented ribbon. Scale bars = 200 µm. (**H**,**I**): Representative transmission electron micrographs showing the auditory nerve fibers in semi-thin sections of the habenula perforata in the cochlear region coding 16–25 kHz from control (**H**), and 2 weeks (**I**) after exposure. (**J**): Quantitative assessment of auditory nerve fibers density in control (blue) and 2 weeks (red) after exposure (*n* = 200 and 400 individual fibers per group and from 4–5 cochleae per each condition). All data are expressed as mean ± SEM, one-way ANOVA test was followed by Dunn’s test.

To determine the contribution of the loss of ribbon synapses in impulse noise-induced reduction of ABR wave-I amplitudes at suprathreshold levels, we examined IHCs by double-labeling presynaptic and postsynaptic structures and 3D confocal imaging analysis [40] in the cochlear regions coding between 6 and 45 kHz. Synapses were identified as juxtaposed presynaptic ribbons and postsynaptic AMPA receptor clusters that were characterized by staining with antibodies against CtBP2 and Homer 1, respectively (Figure 5A–C). Our results showed that control, unexposed ears displayed a broad peak of roughly 15 to 19 ribbons and paired synaptic puncta (synapses) per IHC at cochlear regions tuned to frequencies between 8 and 45 kHz (Figure 5D,E).

One day post exposure, there was a moderate loss of ribbons (Figure 5D) and a more severe loss of synapses (Figure 5E), spanning the cochlear regions from 8 to 45 kHz. The loss reached significance in the 32 and 45 kHz regions (32 kHz: *p* < 0.001, 45 kHz: *p* < 0.05) for synapse counting (Figure 5E), but only at 32 kHz for ribbon counting (*p* < 0.05, Figure 5D). In addition, one day after exposure, a significant increased number of orphan ribbons was observed at 45 kHz (Figure 5D). Two weeks after exposure, the loss of ribbons and synapses was nearly completely recovered, the synaptic counts for the control vs. 2 weeks post exposure are statistically indistinguishable (Figure 5D,E). These results suggest that repair of damaged synapses had taken place, as shown by previous studies [41,42,43].

To examine the ultra-structure of ribbons synapses, we used TEM (Figure 5F,G). In control unexposed IHCs, we often found ribbon synapses which were regular in shape and size. The ribbon bodies were surrounded by a well-organized halo of synaptic vesicles (Figure 5F). Two weeks after exposure, most of the ribbon synapses that we found were presenting electron-dense cores (Figure 5G). In addition, some IHC synapses have immature morphology with double ribbons (left in Figure 5G) which is typically a developmental trait that occurs during the period of cochlear development and during synaptic repair in the post-traumatic period [43,44,45]. By contrast, in most HCs from noise-exposed mice at 2 weeks after, the postsynaptic density was still clearly visible (blue arrows) and so were docked vesicles. Together these results suggest incomplete synaptic regeneration or repair of IHC ribbon synapses which may explain at least partial recovery of the ABR Wave I amplitudes.

Finally, the density of the auditory nerve-fiber terminals in the habenular openings (Figure 5H–J) in both control and noise-exposed mice at 2 weeks after was similar (90.7 ± 1.9 versus 92.9 ± 2.6 fibers per 1000 µm^2^ for control and exposed mice, respectively, Figure 5J).

### 3.6. Oxidative Stress

**Figure 6:**
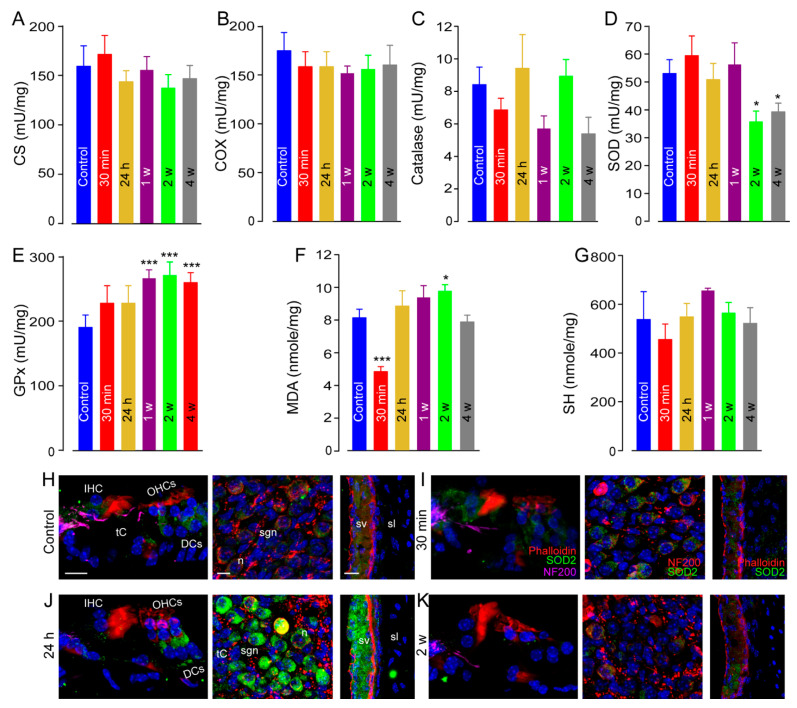
Oxidative stress. (**A**–**G**): Quantitative analysis of citrate synthase (CS), (**A**), cytochrome c oxidase (COX), (**B**), catalase (**C**), superoxide dismutase (SOD), (**D**), and Glutathione peroxidase (GPx), (**E**) activities, and the levels of malondialdehyde (MDA), (**F**) and thiols (SH), (**G**) using spectrofluorochemistry in whole cochlear extracts from control and impulse-noise-exposed cochleae at the different times after exposure (*n* = 16 cochleae per condition). All data are expressed as mean ± SEM, one-way ANOVA test was followed by Dunn’s test. * *p* ≤ 0.05, *** *p* ≤ 0.001, vs. control. (**H**–**K**): Confocal images of transverse cryostat sections of the organ of Corti (left columns), SGNs (mid columns) and stria vascularis (right columns) from the cochleae of control (**H**) and impulse noise-exposed mice at 30 min (**I**), 24 h (**J**), and 2 weeks (**K**) after. Sections were immunolabeled with antibodies against SOD2 (green), and NF 200 (red in mid columns). Phalloidin rhodamine and DAPI were used to label actin and nuclei, respectively. Note that SOD2 immunoreactivity is increased at 24 h after, and reduced at 2 weeks after, exposure, mainly in OHCs, Deiters’ cells (DCs), SGNs, and stria vascularis. tC: tunnel of Corti, sgn: spiral ganglion neuron, sv: stria vascularis, sl: spiral ligament. Scale bars =15 µm.

Mitochondria play a key role in cochlear homeostasis and in maintaining cell function during exposure to sound. To assess the effects of impulse-noise exposure on mitochondrial activity, we measured citrate synthase (CS) activity (a marker of mitochondrial density) and cytochrome c oxidase activity (COX) (complex IV of mitochondrial respiratory chain) in unexposed cochleae and in others at different times after noise exposure. Here, we report no significant differences between the groups (Figure 6A,B).

One of the commonly recognized mechanisms mediating noise-induced cochlear damage is oxidative stress [46]. To test the occurrence of oxidative stress in cochlear tissues after impulse-noise exposure, we assessed the activity of some first-line anti-oxidant enzymes such as catalase (Cat), superoxide dismutase (SOD), and the glutathione peroxidase (GPx), which are recruited to counteract free-radical damage. In addition, lipid peroxidation and protein oxidation were analyzed by measuring the levels of malondialdehyde (MDA) and thiols (SH). Whereas catalase activity was not influenced by noise exposure (Figure 6C), we found that SOD activity was significantly reduced 2 weeks after (*p* < 0.05, Figure 6D). By contrast, the activity of glutathione peroxidase was significantly increased by 1 week after exposure and maintained to 4 weeks after (vs. control: *p* < 0.001, Figure 6E). We also observed that MDA levels were strongly reduced 30 min after sound exposure (vs. control: *p* < 0.001, Figure 6E), whereas no significant difference in the level of SH was found between groups (Figure 6G).

Confocal microscopy observations revealed very strong SOD2 expression in the cytoplasm of the OHCs and their supporting Deiters’ cells, SGNs, and the cells of stria vascularis of the cochleae 24 h after exposure (Figure 6J), compared with control unexposed cochleae (Figure 6H) and 30 min after (Figure 6I). Finally, a drastically reduced SOD2 level was observed in the sensory hair cells, SGNs, and strial cells by 2 weeks after exposure (Figure 6K). Together, these results show that noise exposure elicits oxidative stress.

## 4. Discussion

Firearms and some industrial equipment can generate high levels of impulse noise that may cause traumatic TTS or PTS and/or tinnitus through mechanical injuries of the structures of the middle (e.g., eardrum rupture, disruption of the ossicular chain) [47] and inner ear (e.g., mainly OHC damage) [48,49], as well as metabolic disturbances of the cochlea (e.g., ischemia/reperfusion injury, oxidative stress) [50,51].

### 4.1. Impulse Noise Did Not Induce Eardrum Rupture

It has been suggested that the “threshold” for eardrum rupture in humans is about 185 dB SPL peak [52]. A mouse study demonstrated that exposure to 199 dB SPL peak blasts caused rupture of the tympanic membrane and widespread loss of OHCs in all animals [53]. To assess the effects of impulse noise on cochlear function and morphology, we used an impulse noise with 145 dB SPL peak, 1 impulse/s for 700 impulses. Our results showed no visible eardrum rupture. These results are consistent with DPOAE changes, showing a smaller reduction in the amplitude of DPOAEs than in ABR threshold shifts, since a larger reduction in DPOAEs than ABR threshold shifts would be expected in the case of middle ear damage (middle ear damage affects DPOAE twice).

### 4.2. Reversible Shifts of ABR Thresholds and Reduction of DPOAE Amplitude at Higher, but Not at Lower, Frequencies

To date, relatively little is known about the effects of impulse-noise exposure on the cochlea. A recent study showed that exposure to impulse noise with peak pressures from 160 to 175 dB SPL caused >40 dB TTS, with minimal PTS or HC loss, although often causing a synapse loss of 20–45% in chinchillas [17]. Here, we showed that exposure to impulse noise with peak levels of 146 dB SPL induced ~30 dB TTS with <5 dB PTS only at lower frequencies (4 and 6 kHz), but not at higher frequencies (8 to 32 kHz). These ABR results are consistent with DPOAE assessments showing a very small reduction in DPOAE amplitude, and only at lower frequencies, reflecting an impaired function of the OHCs located in the apical part of the cochlea. SEM evaluation revealed noise-induced, persistent disturbance, revealed in fragmented or fused stereocilia of the OHCs, mainly those located in the cochlear region coding the frequencies from 4 to 16 kHz. Our data are consistent with previous findings in rats, showing that the elevation of ABR thresholds after blast exposure was primarily caused by outer hair cell dysfunction induced by stereociliary bundle disruption [54]. Henderson et al. also demonstrated that exposure to 50 impulses of 166 dB peak SPL induced a median 5 to 15 dB PTS, with all chinchillas having substantial hair-cell lesions [55]. In the present study, complete recovery of the ABR thresholds and DPOAE amplitude in the higher frequencies was consistent with morphological assessments showing no significant hair-cell lesions in the basal part of the cochlea. These data showing the influence of impulse exposure on lower-frequency hearing might infer that the OHCs located in the apical part of the cochlea are more vulnerable to impulse-noise-induced injuries.

### 4.3. Persistent Reduction of ABR Wave-I Amplitude and Elevated Central Gain

Some recent studies in guinea pigs and mice have shown that, for continuous noise exposures, IHC synapses are the most vulnerable elements in the inner ear [11]. A permanent loss of ≥50% of the IHC synapses causes a reduction in ABR wave I amplitude without any elevation of ABR thresholds [11,40]. Here, significant reductions in wave I amplitudes were observed at all frequencies during the acute injury and recovery phases, with little PTS (<5 dB), and that only at lower frequencies (4 and 6 kHz). This small PTS is due to disruption of the OHC stereociliary bundles, mainly in the apical part of the cochlea, and might at least in part explain the reduction in ABR wave I amplitudes in the lower-frequency region. Surprisingly, our impulse-noise paradigm induced ∼30% of IHC synapse loss during acute injury, which then almost recovered by 2 weeks after exposure in all cochlear regions, despite a reduction in ABR wave I amplitude. This mismatch between reduced ABR wave I amplitude and recovery of IHC synapses might be explained by aberrant synaptic reconnection and ribbon morphology, as illustrated by our TEM evaluation of the ribbon synapses. These results are consistent with the data of Song et al., [56] showing coding deficits in hidden hearing loss induced by noise. Finally, we observed that decreased ABR wave I amplitude is associated with an increased Wave V amplitude, suggesting that a decrease in the input to the auditory central nervous system induced a compensatory increase in central gain. This latter might be a causal factor of noise-induced tinnitus and hyperacusis.

### 4.4. Oxidative Stress

Numerous authors have reported that noise exposures may cause subsequent secondary cochlear lesions through oxidative stress, an inflammatory process [26]. Cells are armed against oxidative stress and are endowed with robust anti-oxidant defenses to counteract excessive ROS/RNS production via the activity of anti-oxidant enzymes such as manganese superoxide dismutase (MnSOD/SOD2), copper/zinc superoxide dismutase (Cu/Zn SOD/SOD1) or catalase, GPX (glutathione peroxidase) [57]. 

It has been reported that blast exposure that is known to cause the ear and lung injuries that can induce oxidative stress in the lung characterized by anti-oxidant depletion, lipid peroxidation and hemoglobin oxidation in rats [58]. Anti-oxidant treatments reduced hemoglobin oxidation and lipid peroxidation [58] as well as impulse noise-induced hearing loss in rats [59]. In addition, administration of 4-[2-aminoethyl] benzenesulfonyl fluoride, an inhibitor of NADPH oxidase activation onto the round window membrane of the impulse noise-exposed chinchillas reduced noise-induced permanent threshold shift [60]. Overall, these results suggest a link between impulse noise-induced oxidative stress and cochlear cell damage.

In this study, we demonstrate that oxidative stress was induced in the cochlea after impulse-noise exposure, as demonstrated by the early increase in SOD2 expression, together with a significantly reduced MDA level, suggesting the activation of anti-oxidative defense mechanisms in the cochlea under stressful conditions. At a later stage (from 1 to 2 weeks after exposure), the appearance of oxidative stress in the cochlea was characterized by markedly reduced anti-oxidant SOD activity and SOD2 expression, together with an overproduction of lipid peroxidation (MDA). These results indicate ROS-induced, progressive oxidative damage. On the other hand, a persistent and significant increase in the activity of GPXs was observed from 1 to 4 weeks, which is widely accepted as a stress “enzyme” and proposed as a biomarker for sublethal metal toxicity in plants [61]. Altogether, these results indicate that even though our impulse-noise paradigm caused only very small PTS at lower frequencies, it induced persistent oxidative stress in the cochlear cells.

## 5. Conclusions

In the present study, we have shown that a moderate impulse-noise exposure caused an elevation of ABR thresholds and a reduction in DPOAE amplitude immediately after exposure, which then returned to normal at the higher frequencies 2 weeks later. Only a very small level of PTS at the lower frequencies (4 and 6 kHz) was seen 4 weeks later. The small PTS was due to a permanent disturbance of the stereociliary bundle of OHCs located in the apical part of the cochlea. Even though the ABR threshold shifts have completely or almost completely recovered, a permanently reduced amplitude of the ABR wave I was observed for all frequencies tested even four weeks after exposure. The permanent reduction in the amplitude of wave I is despite a complete recovery of the number of synapses. This could be explained by a morphological modification of the regenerated synapses revealed with TEM evaluation. Finally, we observed a persisting increase in the levels of oxidative stress up to 2 weeks after noise exposure. These results highlight the potential roles of oxidative stress in impulse-noise-induced damage to the cochlear sensory neural cell resulting in hidden hearing loss.

## Data Availability

The data is contained within the article.

## Supplementary Materials

The following are available online at https://www.mdpi.com/article/10.3390/antiox10121880/s1. Figure S1. ABR audiograms tracking changes of thresholds for a period of 4 weeks.
