# Supplementary material for: Impulse Noise Induced Hidden Hearing Loss, Hair Cell Ciliary Changes and Oxidative Stress in Mice
Source: Antioxidants (Basel). 2021 Nov 25;10(12):1880. doi: 10.3390/antiox10121880 (PMC8698479; doi:10.3390/antiox10121880)
Supplement: Supplementary file 1 [file antioxidants-10-01880-s001.zip › antioxidants-1443735-supplementary.pdf]

Supplementary file 1

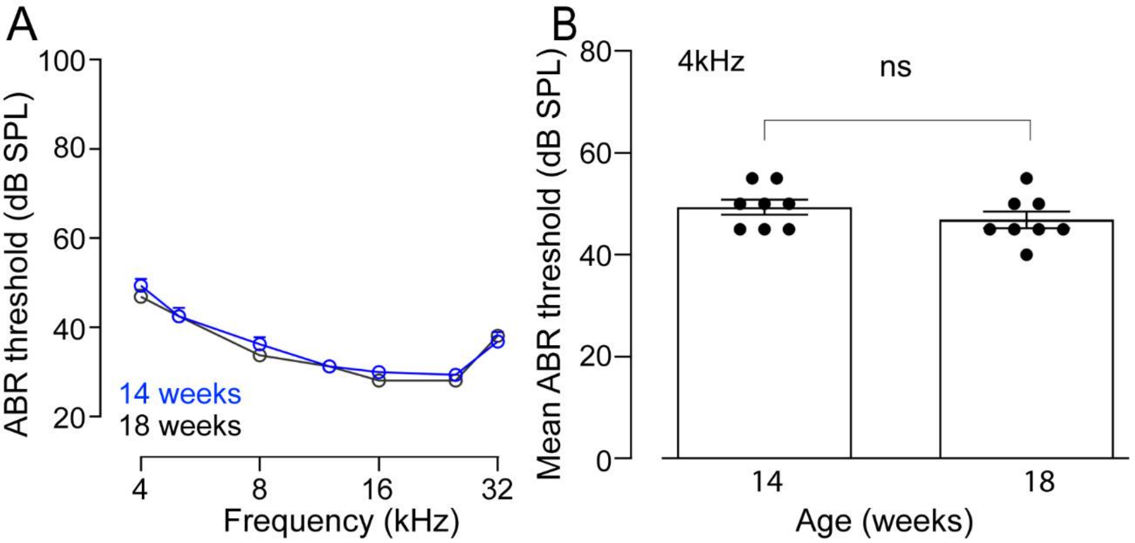

Figure S1. ABR audiograms tracking changes of thresholds for a period of 4 weeks

A: ABR thresholds recorded in the same mice at 14 weeks of age (blue) and 18 weeks of age (black). Note no changes in ABR thresholds recorded in the mice at 14 weeks of age compared with those recorded at 18 weeks of age. **B**: Mean ABR thresholds at 4 kHz for these two evaluation ages. All data are expressed as mean ± SEM (n=4 mice, 8 ears), paired t test (14 vs 18 weeks).
